# Editorial: Cardiorenal dysregulation in endocrine disorders: innovative mechanisms and therapeutic interventions

**DOI:** 10.3389/fphar.2023.1275919

**Published:** 2023-08-25

**Authors:** Noha M. Shawky, Eman Soliman, Abdel A. Abdel-Rahman, Samar Rezq

**Affiliations:** ^1^ Department of Pharmacology and Toxicology, Mississippi Center of Excellence in Perinatal Research, Women’s Health Research Center, Cardiovascular Renal Research Center, University of Mississippi Medical Center, Jackson, MS, United States; ^2^ Department of Pharmacology and Toxicology, Faculty of Pharmacy, Zagazig University, Zagazig, Egypt; ^3^ Department of Biomedical Sciences and Pathobiology, Virginia Tech, Blacksburg, VA, United States; ^4^ Department of Pharmacology and Toxicology, Brody School of Medicine, East Carolina University, Greenville, NC, United States

**Keywords:** cardiovascular, renal, endocrine, non-coding RNA, autophagy

Endocrine disorders increase the risk of cardiovascular (CV) and renal abnormalities, leading to end-organ damage and chronic comorbidities. However, diagnosing and treating these complications present challenges due to limited therapeutic options. Therefore, further research is essential to understand the novel molecular mechanisms underlying cardiorenal dysregulation caused by endocrine disorders and to explore innovative therapeutic approaches. In this editorial commentary, we summarize four articles that offer insights into managing cardiorenal dysregulation in the context of endocrine disorders. The aim of this commentary is to highlight proposed approaches for preventing, delaying progression, or effectively treating this condition.

Diabetic nephropathy (DN) is a common diabetes-associated microvascular complication, contributing to over 50% of end-stage renal disease cases. Investigated the role of mitophagy, selective degradation of damaged mitochondria by autophagy, in DN progression. Using db/db mouse model and complementary human podocytes *in vitro* assays, Zheng et al. uncover the impact of Src and FUNDC1-associated mitophagy in diabetic renal damage. The authors reported that activation of Src, a non-receptor tyrosine kinase, phosphorylates FUNDC1, inhibits FUNDC1-mediated mitophagy, and therefore promotes renal damage in models of diabetes. Therefore, activating mitophagy via Src inhibition emerges as a potential strategy for DN treatment.

Myocardial hypertrophy is a pathological condition that occurs in many CV diseases (CVD) and typically precedes the onset of heart failure. Different endocrine factors such as thyroid hormones, insulin, insulin-like growth factor 1, angiotensin II (Ang II), endothelin, catecholamines, estrogen, among others constitute important stimuli for triggering and/or preserving myocardial hypertrophy (Takano et al., 2020). Thus, numerous endocrine disorders manifested as changes in the local environment or multiple organ systems are important in the context of progression from myocardial hypertrophy to heart failure. The study by describes the effects of Hirudin, a potent direct thrombin inhibitor anticoagulant, which is isolated from the saliva of leech (Dong et al., 2016), on myocardial hypertrophy.

Utilizing an advanced network pharmacology method, which integrates system biology and computer bioinformatics technology, Liu et al. found that through its good binding ability, hirudin may inhibit myocardial hypertrophy by regulating: (1) the phosphatidylinositol 3-kinase (PI3K)/protein kinase B (AKT) signaling pathway and (2) key targets such as signal transducer and activator of transcription (STAT)-3, interleukin (IL)-6, and mitogen-activated protein kinase (MAPK)-1. Using H9c2 cells and neonatal rat cardiomyocytes, the authors investigated the inhibitory effects of hirudin on Ang II-induced cellular hypertrophy. They found that downregulation of STAT-3, IL-6 and MAPK-1 mRNA and inhibition of the phosphorylation of STAT3 and MAPK-1 likely explain the hirudin-mediated inhibition of Ang II-induced hypertrophy.

Myocardial ischemia-reperfusion injury (MIRI) is a major contributor to adverse CV outcomes following acute myocardial infarction (Frank et al., 2012). MIRI was found to be regulated by various endocrine factors such as insulin (Ji et al., 2010), adiponectin (Wang et al., 2010), estrogen (Zhai et al., 2000a; Zhai et al., 2000b), renin-angiotensin system (Zughaib et al., 1993; Grobecker et al., 1993) and others. The review by Wang et. al. described the regulatory role of non-coding RNAs (ncRNAs) in the pathogenesis of myocardial ischemia-reperfusion injury (MIRI). The article provides valuable insights into how ncRNAs influence the outcome of MIRI by modulating autophagy, which impacts the severity of MIRI via selective or non-selective degradation of cytoplasmic components.

NcRNAs regulate various steps of the non-selective autophagy process, including autophagy induction, nucleation of the autophagosome, extension of the autophagosome, and fusion of the autophagosome with the lysosome. Several microRNAs (miRNAs or miRs, a form of ncRNA) target the expression of genes involved in mammalian target of rapamycin (mTOR)-mediated autophagy regulation during myocardial ischemia. For instance, regulation of miR-30a, miR-208a, miR-494 and miR-384, miR-29c modulate PI3K/AKT/mTOR pathway resulting in autophagy-mediated cardioprotective effects. Other miRNAs (e.g., miR-429, miR-139-5p, miR-206, and miR-300) activate mTOR, and thereby inhibit autophagy, by regulating AMPK pathway. Inversely, miR-99a can directly target mTOR and downregulate the mTOR/P70S6K signaling pathway to improve MIRI. In addition, long ncRNAs (lncRNAs; such as MALAT1, PVT1, AK088388, H19, RMRP, NEAT1, and AK139328) regulate autophagy by acting as competitive endogenous RNAs or by directly binding to transcription factors. Selective autophagy of damaged mitochondria (mitophagy) in cardiomyocytes is also regulated by miRNAs. MiR-410, downregulated during hypoxia/reoxygenation (H/R) in human adult cardiomyocytes, attenuates mitophagy, worsening MIRI by targeting HMGB1. MiR-302a-3p suppression promotes mitophagy through FOXO3, enhancing mitochondrial function and MIRI mitigation. Emerging evidence suggests that ncRNAs like circular RNA (circRNA) ACR and circPAN3/miR-421 regulate the expression of Pink1 (a pivotal mitophagy regulator) affecting mitophagy and MIRI outcomes. Taken together, the involvement of miRNAs, lncRNAs, and circRNAs in autophagy regulation suggests that ncRNAs are expected to be a new biomarker for diagnosing/predicting MIRI and a druggable target for mitigating MIRI.

The review by Xu et al. highlights the significance of a particular miRNA (miR-132), an essential regulatory RNA expressed in the CV system, in the regulation of various acute and chronic cardiorenal disorders. These disorders include conditions related to endocrine disorders, such as ischemic CVD, heart failure, diabetes mellitus, and acute renal injury. The review article presents a comprehensive analysis of miR-132’s role in cardiorenal pathophysiology, showcasing multiple mechanisms involved, such as myocardial hypertrophy, fibrosis, apoptosis, angiogenesis, calcium handling, neuroendocrine activation, and oxidative stress. Furthermore, the article provides valuable insights into both preclinical and clinical studies that support the potential of miR-132 as a promising therapeutic for treating CVD. These studies involve either downregulation or overexpression of miR-132 to achieve therapeutic effects.

In conclusion, the articles included in our Research Topic highlight a number of novel mechanisms involved in cardiorenal dysregulation in endocrine disorders. Insights into hirudin’s protective potential against myocardial hypertrophy, mitophagy regulation in DN, and ncRNA as a master regulator in CVD offer novel directions for therapeutic innovation. Further preclinical research and clinical trials are warranted to translate these findings into personalized strategies to manage cardiorenal dysregulation in patients with endocrine disorders.
